# Life cycle environmental and economic impacts of nutrient management in small community lagoon wastewater systems

**DOI:** 10.1016/j.scitotenv.2026.181978

**Published:** 2026-06-27

**Authors:** Denis Ruto, Corbin Vincent, Ziya Jang, Pablo Cornejo, Harold Leverenz, Steffen Mehl, Kevin Orner

**Affiliations:** a Wadsworth Department of Civil and Environmental Engineering, West Virginia University, 1306 Evansdale Drive, PO Box 6103, Morgantown, WV, 26506-6103, USA; b Department of Civil and Environmental Engineering, California State University, Chico, 400 West First Street, Chico, CA, 95929, USA; c Department of Civil and Environmental Engineering, University of California, Davis, One Shields Avenue, Davis, CA, 95616-5270, USA

**Keywords:** Stabilization ponds, Carbon footprint, Eutrophication, Sustainability assessment, Nutrient removal

## Abstract

Excess nitrogen and phosphorus discharges from wastewater lagoons pose persistent challenges for small communities, potentially contributing to eutrophication, and regulatory non-compliance. While lagoons remain popular due to operational simplicity and cost effectiveness, they often achieve limited nutrient removal relative to some alternative technologies. This study evaluated nine nutrient management scenarios for small community lagoons in the U.S. using life cycle assessment and life cycle cost analysis. Scenarios included a baseline clay-lined facultative lagoon, a membrane-lined facultative lagoon, an aerated lagoon with maturation pond, and six aerated lagoon–maturation pond configurations with either biodomes, iron-dosed upflow reactor, packed-bed biofilm reactor, moving bed biofilm reactor, constructed wetland, or an algal pond. Environmental impacts were assessed as global warming potential (GWP) and freshwater and marine eutrophication potential (FEP and MEP, respectively), while economic performance was measured as present worth of costs (PWC) over a 20-year system lifetime. Baseline lagoons were cost effective ($0.20–$0.30/m^3^) but exhibited the highest GWP (~3 kg CO_2_ eq/m^3^), FEP (~0.015 kg P/m^3^), and MEP (~0.009 kg N/m^3^). Packed-bed reactor and iron-dosed upflow reactor scenarios achieved 70% reductions in both FEP and MEP, with moderate GWP (0.8–1.0 kg CO_2_ eq/m^3^), though at high costs ($0.86–$1.19/m^3^). Sensitivity and uncertainty analyses identified effluent nutrient concentrations, design flow, and capital costs as dominant factors influencing outcomes. Overall, this study offers a robust framework for comparing treatment alternatives beyond upfront costs alone, guiding the selection of environmentally responsible, economically viable, and locally appropriate nutrient management solutions for small communities.

## Graphical Abstract

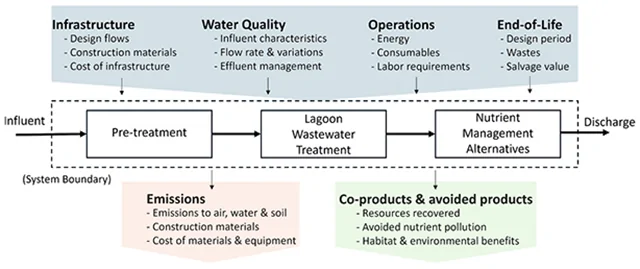

## 1. Introduction

Elevated concentrations of nitrogen and phosphorus in water bodies can have severe consequences for both human and aquatic health. High ammonia levels are toxic to aquatic organisms, while excess nitrate and phosphorus contribute to eutrophication in surface waters and groundwater contamination, posing serious public health risks (Archer and O'Brien, 2005). Managing the nitrogen cycle and providing access to clean water are among the 14 Grand Challenges for Engineering identified by the U.S. National Academy of Engineering, underscoring their importance in safeguarding freshwater, marine, and groundwater resources (National Academy of Engineering, 2008). A major pathway through which nutrients enter aquatic ecosystems is via wastewater discharges (U.S. EPA, 2011). Consequently, the removal or recovery of nutrients has become an important issue in the 21st century.

Lagoon wastewater treatment systems, also known as waste stabilization ponds, are engineered ponds designed to utilize natural processes to remove organics, suspended solids, and nutrients from wastewater (von Sperling, 2007). These lagoon systems typically fall into three categories: anaerobic, facultative, and aerated. They can be used independently, in sequence, or in tandem with other technologies such as constructed wetlands (Middlebrooks et al., 1982). Due to their low energy requirements, operational simplicity, and cost-effectiveness, lagoon systems are especially well-suited for small communities (those with fewer than 10,000 residents) (Crites et al., 2014).

In the United States, small communities utilizing lagoons often face unique wastewater treatment challenges, including limited technical expertise, insufficient funding for capital and operational expenses, and growing pressure to meet tightening regulatory standards (U.S. EPA, 2022a). Although lagoon systems are generally effective at removing organic matter and suspended solids, they typically achieve less than 50% removal efficiency for ammonia, total nitrogen (TN), and total phosphorus (TP) across different configurations (von Sperling, 2007). A 2022 study by the U.S. EPA found that nearly two-thirds of lagoons failed to meet effluent standards, including nutrient levels (U.S. EPA, 2022b).

To improve nutrient removal in lagoons, several enhancement strategies have been proposed for lagoon systems in small communities. For nitrogen removal, in-lagoon aeration and mixing can promote nitrification, while adding advanced units such as membrane bed biofilm reactors can enable full nitrogen removal via nitrification and denitrification (Bhattacharya and Mazumder, 2021). For phosphorus, chemical precipitation using agents like aluminum sulfate is common. Biofilters have also emerged as a promising option for combined phosphorus removal and recovery (Bunce et al., 2018).

It is worth noting that for some communities in certain regions, especially in arid and semi-arid climates where evaporation exceeds precipitation, low- and no-discharge lagoon systems are increasingly encouraged. These systems can reduce or eliminate surface water nutrient discharges and may offer advantages in terms of lifecycle cost, regulatory compliance, and environmental impacts (Rodewald et al., 2023; U.S. EPA, 2011). However, such systems were not considered in the study, which focused on nutrient management strategies for continuously discharging lagoon systems subject to the National Pollutant Discharge Elimination System (NPDES) permitting requirements.

While performance evaluations of these nutrient removal technologies are important, it is equally critical to assess their environmental and economic sustainability across the full life cycle. Life cycle assessment (LCA) provides a structured approach to quantify environmental impacts such as global warming potential and eutrophication across the service life of wastewater treatment systems. For instance, Lin et al. (2016) found that ion exchange, when compared to anammox and conventional nitrification-denitrification systems, had the lowest impacts in several midpoint categories, including ozone depletion and abiotic resource use. Similarly, Vineyard et al. (2021) reported that electrodialysis outperformed biological nitrogen removal methods in reducing global warming and acidification per kg of ammonium removed.

Despite a growing body of LCA literature on wastewater treatment systems, most studies focus on large urban systems with flow rates exceeding 3785 m^3^/day (10,000 population equivalents) (Lam et al., 2020; Ontiveros and Campanella, 2013). Research specific to lagoons used as the main secondary treatment technology remains limited. When lagoons are included in LCA studies, they are often examined as part of larger treatment trains or compared with other secondary treatment technologies. For example, Thompson et al. (2022) compared three lagoon configurations with a mechanical treatment system under assumptions of no discharge or intermittent discharge, where sufficient storage was available to allow effluent release only when NPDES limits were met. In contrast, this study focused on continuously discharging lagoon systems and evaluates alternative nutrient management strategies under typical NPDES permitting conditions for small communities. Moreover, some of these studies were conducted in tropical settings such as Bolivia and Costa Rica, limiting their relevance to small communities in the United States (Cornejo et al., 2013; Orner et al., 2021).

Given the financial constraints that small communities often face, evaluating the cost-effectiveness of nutrient management strategies is critical to ensuring sustainable implementation. However, most wastewater cost studies disproportionately focus on large-scale systems and are often derived from generalized cost curves that aggregate different design flows. These approaches often concentrate on individual unit processes and inadvertently overlook the integrated nature of treatment systems (U.S. EPA, 2021).

Life cycle cost analysis (LCCA) provides a comprehensive framework by capturing the full range of economic impacts, including capital, operational, maintenance, and end-of-life costs, associated with complete treatment trains (Abyar and Nowrouzi, 2023). By applying LCCA, decision-makers can better understand long-term cost trade-offs and identify strategies that balance regulatory compliance, environmental performance, and affordability. Thus, there is a clear need for further research to assess both the environmental and economic life cycle impacts of nutrient management strategies tailored to lagoon wastewater systems in small communities.

Accordingly, this study evaluated the sustainability of nutrient management strategies for lagoon wastewater systems in small communities by examining both their environmental impacts and cost implications across the full life cycle, including construction, operation, and end-of-life stages. Nine nutrient management scenarios serving less than 10,000 population equivalents (P.E.) were evaluated. Three scenarios were based on real case studies with design flows of 0.04, 0.2, and 0.4 MGD (400, 2000 and 4000 P.E., respectively) while the remaining six alternatives were modeled using use a representative flow of 1 MGD (10,000 P.E.), using a generic site and design conditions across scenarios. All results were normalized to 1 m^3^ of treated wastewater for consistent comparison. Site-specific conditions and local regulations that may impact design considerations were not explicitly accounted for in this analysis.

All nine scenarios were assessed using LCA to quantify carbon footprint (as global warming potential) and eutrophication potential. In parallel, economic feasibility was evaluated through LCCA, accounting for capital expenditures, operating and maintenance costs, and decommissioning expenses. Together, these assessments provide an improved understanding of environmental and economic trade-offs, generating insights to inform decision-makers in small communities seeking to comply with stricter nutrient discharge regulations cost-effectively, minimizing environmental harm.

## 2. Methods

### 2.1. Life cycle assessment (LCA)

The environmental impacts of upgrading lagoon systems with different nutrient management alternatives were evaluated using LCA following ISO 14040 guidelines. The LCA followed the standard four-phase framework: (1) goal and scope definition, (2) life cycle inventory analysis, (3) impact assessment, and (4) interpretation (ISO, 2006; U.S. EPA, 2021).

#### 2.1.1. Goal and scope definition

**Fig. 1.**
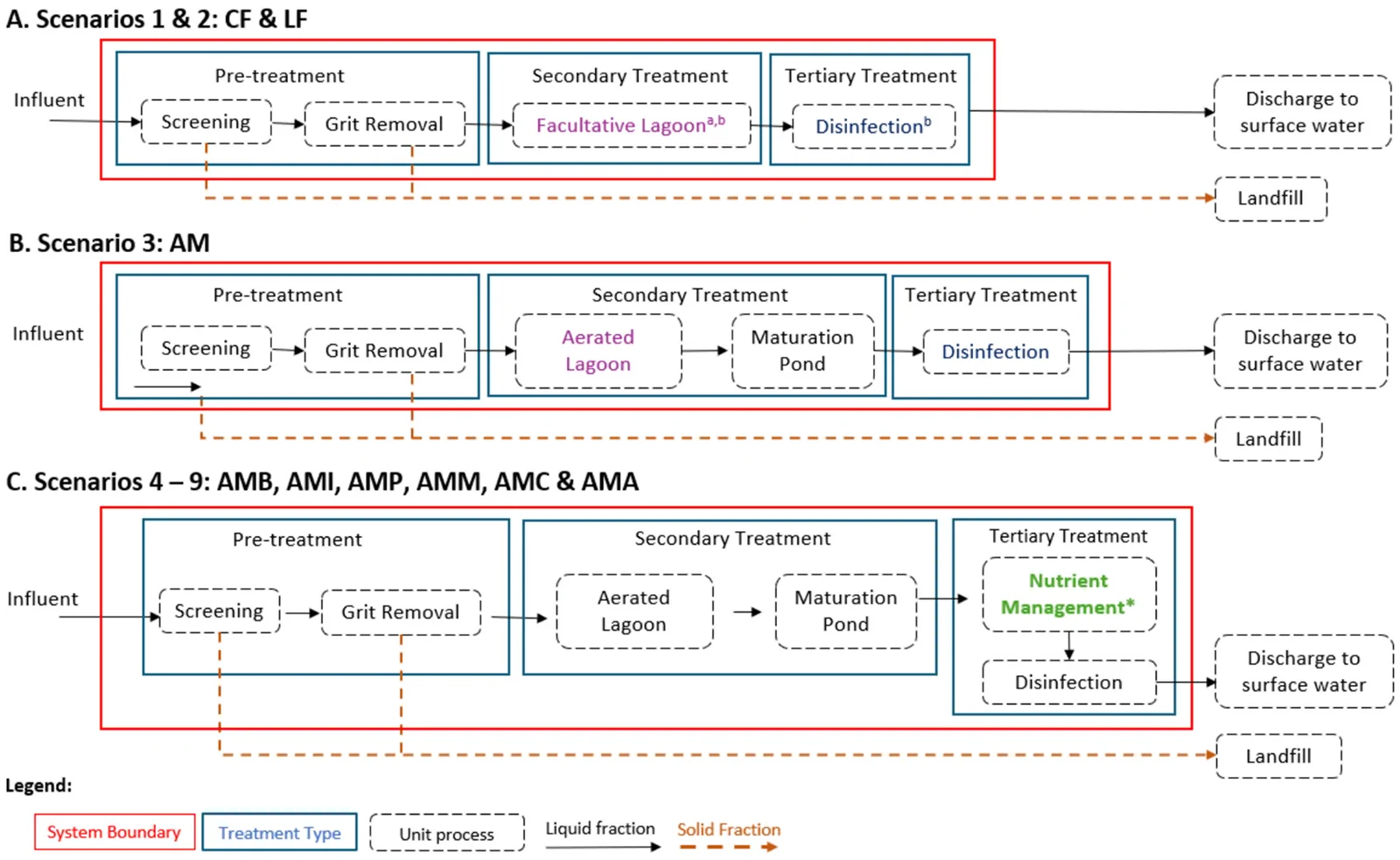
Process flow diagrams of 9 treatment scenarios considered in this study. A represents Scenarios 1 & 2: clay-lined facultative (CF) lagoon and membrane-lined facultative lagoon (LF), respectively. Footnote ‘a’ represents facultative lagoon with clay liner and no disinfection, while ‘b’ facultative lagoon with a membrane liner and disinfection. B represents Scenario 3 Aerated Lagoon + Maturation Pond (AM). C* represents scenarios 4–9, each combining AM with one of six nutrient management technologies, i.e., biodomes (AMB), iron-dosed UASB (AMI), packed-bed reactor (AMP), MBBR (AMM), constructed wetland (AMC), and algal-based system (AMA).

The goal of this LCA was to evaluate the carbon footprint and eutrophication potential of nine nutrient management alternatives for lagoon wastewater treatment systems in small communities. The scope covered the construction, operation, and end-of-life phases of each treatment scenario, with the system boundary encompassing pretreatment, secondary treatment, and tertiary treatment including nutrient removal and disinfection as illustrated in Fig. 1.

#### 2.1.2. Scenario descriptions

This study assessed nine nutrient management scenarios: (1) clay-lined facultative lagoon (CF); (2) membrane-lined facultative lagoon (LF); (3) aerated lagoon + maturation pond (AM); and six upgraded configurations building on AM by integrating additional treatment technologies, i.e., (4) biodomes (submerged, dome-shaped biofilm reactors) (AMB), (5) iron-dosed upflow anaerobic sludge blanket (UASB) reactor (AMI), (6) submerged, aerated packed-bed biofilm reactor with woodchips (AMP), (7) moving-bed biofilm reactor (MBBR) (AMM), (8) subsurface horizontal constructed wetland (AMC), and (9) high-rate algal pond (AMA) (Fig. 1).

The constituent technologies of these scenarios were selected following a comprehensive literature review by the authors, which identified them as among the most feasible nutrient management strategies in small, municipal lagoon systems. Scenario 1 (CF) was designated as the baseline, representing the most basic treatment configuration still in use. From there, the scenarios reflect a progressive sequence of upgrades, starting with low-cost enhancements such as membrane lining (LF) and aeration (AM), and advancing toward increasingly complex interventions. These included enhanced biological systems (AMB, AMM, AMP), nature-based solutions (AMC), phosphorus recovery through vivianite precipitation (AMI), and algae-based resource recovery (AMA).

Effluent water quality levels were scenario-specific and reflected the expected performance of each treatment configuration and technology. For scenarios CF, AM, and AMB, effluent concentrations were based on measured data from the corresponding case study sites, while values for the remaining scenarios were derived from peer-reviewed literature and U.S. EPA's data monitoring reports for comparable treatment trains and operating conditions (US EPA, 2024; U.S. EPA, 2011).

#### 2.1.3. Life cycle inventory

Life cycle inventory (LCI) data were compiled for each scenario across three life stages: construction, operations, and end-of-life. All data were normalized to a functional unit of 1 cubic meter of treated wastewater over a 20-year system lifetime, reflecting typical desludging and upgrade cycles in municipal lagoon systems (Middlebrooks et al., 1982). While service life can vary between 20 and 40 years depending on maintenance practices and growth pressures, a 20-year horizon was adopted for comparability across scenarios (Ghangrekar, 2022). A summary of the inventory items across the life cycle stages for each scenario is presented in Table 1.

Detailed flow rates, effluent water quality assumptions, and life cycle inventory data for all scenarios are provided in Table S1 in Supplementary Information. Differences in influent flow and effluent concentrations reflect the use of measured data for case-study systems and literature-based performance values for technology-specific treatment configurations, ensuring realistic representation of each scenario. For CF, AM, and AMB, influent and effluent quality data were obtained through field sampling, while infrastructure data were derived from as-built drawings. For the remaining scenarios, data were supplemented from vendor case studies and published literature.

The construction phase incorporated materials such as HDPE for liners, biodomes, and biomass carriers, PVC and polyethylene piping, and reinforced concrete. Excavation and installation activities were included with separate fuel entries, as well as sand and gravel used as filling materials. In the AMI scenario, iron filings were modeled as an input replaced every four years.

The operation and maintenance phase captured ongoing inputs and outputs over the functional lifetime of each alternative. These included chemicals used for disinfection across all scenarios and precipitation agents in AMP and AMA. Electricity use was included, modeled on the average U.S. energy mix of 43% natural gas, 21% renewables, 19% nuclear, and 16% coal (U.S. EIA, 2024). Emissions included releases to air: biogenic carbon dioxide, methane, CO_2_ from flared methane, and nitrous oxide, as well as nutrient discharges to water (ammonia, total nitrogen and phosphorus). Air emissions were estimated using IPCC (2019) methods based on influent BOD concentrations, while nutrient concentrations were derived from field sampling and literature sources. Although past studies often excluded biogenic emissions, these were included here due to their relevance to wastewater systems (Bare, 2011; IPCC, 2006). Harvested algae was considered a co-product in AMA, while screenings and grit were included as solid waste outputs.

The end-of-life phase encompassed decommissioning activities such as desludging, excavation of reactor basins, and site backfilling. All previously installed materials, including HDPE liners, piping, and concrete, were classified as waste and inventoried accordingly. Energy use for decommissioning was included, as well as the disposal of 20 years of accumulated sludge and spent filling materials.

#### 2.1.4. Data quality assessment

Data quality was assessed using criteria adapted from the pedigree matrix approach developed by Weidema and Wesnæs (1996) and subsequently adopted in Ecoinvent and U.S. EPA life-cycle inventory guidance (Edelen and Ingwersen, 2016; Weidema et al., 2013; Weidema and Wesnæs, 1996). Five indicators were evaluated for each major dataset: reliability, temporal representativeness, geographical representativeness, technological representativeness, and completeness. The data quality goals (Table S2) and pedigree matrix criteria used for scoring (Table S3) are provided in the Supplementary Information.

Table 2 summarizes the data quality assessment results of major datasets used in the LCA and LCCA, with scores ranging from 1 (high quality) to 5 (lowest). The highest data quality scores were assigned to facility-specific operational and cost data, including flow rates, influent and effluent water quality, electricity consumption, labor, and chemical costs, because these data were obtained directly from facility records, monitoring programs, and public utility reports. Construction materials, transportation, vendor quotes, RSMeans cost estimates, and background inventories were assigned good quality ratings due to their broad industry acceptance and relevance to the evaluated systems, although they were not always site-specific. Lower scores were assigned to air emission inventories and resource recovery revenues because they relied on literature-derived emission factors, average removal performance, market assumptions and future demand estimates, resulting in greater uncertainty and reduced site specificity. Under the pedigree matrix framework, datasets derived from the same source, such as electricity consumption and electricity costs, received similar ratings (Edelen and Ingwersen, 2016).

No inventory datasets were assigned low or very low-quality ratings (scores of 4 or 5), indicating that the inventory data were generally of good to high quality and suitable for comparative environmental and economic assessment.

#### 2.1.5. Life cycle impact assessment and interpretation

Life cycle impact assessment (LCIA) was conducted using SimaPro 9.5 to compile life cycle inventories, link foreground processes with background systems, and calculate midpoint environmental impacts. Background processes (e.g., electricity generation, material manufacturing, and transportation) were modeled using the Ecoinvent database (version 3.9.1, cut-off system model) (Wernet et al., 2016). Two midpoint impact categories were evaluated: global warming potential (GWP) and eutrophication potential (EP). GWP was calculated using IPCC, 2019 characterization factors with a 100-year time horizon and reported as kilograms of CO_2_ equivalents per cubic meter of treated wastewater (IPCC, 2019). Eutrophication potential was evaluated separately for freshwater and marine environments using TRACI 2.2 characterization factors, expressed as kilograms of phosphorus equivalents (kg P eq) for freshwater eutrophication potential (FEP) and kilograms of nitrogen equivalents (kg N eq) for marine eutrophication potential (MEP), reflecting the dominant limiting nutrients in the respective receiving water bodies (Bare, 2011; Henderson et al., 2021). Details on the effluent quality used and discussion on variability observed can be found in the Supplementary Information.

### 2.2. Life cycle cost analysis

To complement the environmental assessment, a LCCA was performed to evaluate the long-term economic performance of each scenario over a 20-year horizon, consistent with the LCA. The analysis included capital expenditures, annual operating costs for energy, chemicals, labor, solids handling, and laboratory services, and maintenance activities. Revenues from resource recovery were also incorporated as benefits, offsetting total annual costs where applicable (Hall et al., 2022).

Capital cost estimates were obtained from a combination of vendor quotes and RSMeans construction cost data. Vendor quotes provided technology-specific pricing for specialized components, while RSMeans offered standardized estimates for materials, labor, and installation. For the CF, AM, and AMB scenarios, site-specific cost data for labor, electricity, chemicals, and equipment were obtained directly from facility operators and state public service commission reports, thereby grounding the analysis in real-world conditions.

Present Worth of Costs (PWC) of each scenario was calculated as the main economic performance metric, normalized to dollars per cubic meter of treated wastewater ($/m^3^). PWC accounts for the total life cycle cost in present-value terms, making it a robust framework for comparing alternatives over time (Huang et al., 2020). A discount rate of 6% was applied, consistent with standard practice in infrastructure planning (Cicekalan et al., 2023). Detailed cost data used, and sources are provided in Supplementary Tables 1 and 5.

### 2.3. Sensitivity analysis

A sensitivity analysis was conducted to evaluate the robustness of the model results by identifying inputs with the greatest influence on the outputs. Variables contributing less than 5% to overall impacts were excluded (Salciccioli et al., 2016). The remaining inputs were independently varied by ±20%, while all others were held constant (Jaczynska et al., 2025). Construction parameters tested included plastic liners, excavation, concrete use, and design flow rate. Operational parameters included biogenic carbon dioxide, effluent nitrogen, effluent phosphorus, and treatment energy. End-of-life parameters included waste concrete, waste liner, and accumulated sludge. Sensitivity factors were calculated as the absolute difference between adjusted and baseline impact scores, divided by the baseline value, with larger values indicating stronger influence. This analysis highlighted which inputs most significantly drive variation in environmental performance (Cornejo et al., 2013).

### 2.4. Uncertainty analysis

Uncertainty in environmental and economic performance was evaluated using python-based Monte Carlo simulations in Microsoft Excel with 10,000 iterations per treatment alternative. For environmental parameters (GWP, FEP, and MEP), normal distributions were used based on minimum and maximum ranges from the life cycle inventory (Chowdhury, 2012). Cost parameters were modeled using lognormal distributions to capture their non-negative, right-skewed nature (Bashar et al., 2018). Design flow and analysis period were treated as stochastic input parameters and modeled using normal distributions centered on their baseline values to represent variability around nominal design assumptions, while the interest rate was modeled using a uniform distribution to reflect equal likelihood across a plausible range (Chowdhury, 2012; Ghangrekar, 2022). Uncertainty was presented in form of error bars representing ±1 standard deviation in the resulting graphs.

For clarity, the ±20% variation applied to design flow in the sensitivity analysis was a separate deterministic test used to evaluate the influence of moderate changes in flow on model results and scenario ranking and was not part of the Monte Carlo uncertainty framework.

### 2.5. Selecting suitable technology

To rank the alternatives, each metric GWP, FEP, MEP, and PWC was first normalized to a common scale to enable meaningful comparison across different units and magnitudes. The normalized values were then aggregated to generate an overall impact score for each alternative, with lower scores indicating better combined environmental–economic performance, following the methodology adopted from Hall et al. (2022).

Normalization was performed using min–max scaling:

(1) $$x_{i,j}^{*}=\frac{x_{i,j}-\text{min}(x_{j})}{\max(x_{j})-\text{min}(x_{j})}$$

where *x_i,j_* is the value of indicator *j* for alternative *i*, and $x_{i,j}^{*}$ is the corresponding normalized value (Hall et al., 2022). This transformation rescales each indicator to a dimensionless range between 0 and 1, where 0 represents the best-performing alternative (lowest impact or cost) and 1 represents the worst-performing alternative for that indicator. Following normalization, a composite impact score was calculated for each scenario by summing the normalized values across all four indicators, with each indicator contributing equally to the total score. Scenarios were then ranked in ascending order of the composite score to identify the most suitable treatment technologies. The results were further visualized using radar plots to facilitate comparison of trade-offs among scenarios.

### 2.6. Assumptions

This study assumed dry-weather design flows representative of typical small-community wastewater systems and did not explicitly account for elevated influent volumes due to infiltration and inflow (I&I). Specifically, per-capita flows were assumed to be below levels typically associated with significant I&I impacts. In practice, many small systems experience substantial I&I, which can affect hydraulic loading, treatment performance, infrastructure sizing, energy use, and life-cycle costs (WEF, 2010). Technology resilience to I&I also varies across treatment configurations and may influence optimal system selection (U.S. EPA, 2011). While flow rate was examined in the sensitivity analysis, a detailed evaluation of I&I impacts, and system resilience was beyond the scope of this study and represents an important area for future research.

All scenarios were assumed to meet U.S. EPA effluent standards over a 20-year lifetime and to operate under steady-state conditions with constant influent characteristics, stable treatment performance, no capacity expansion, and no system failures or extreme events. Assumptions influencing material, energy, emissions, and solids inventories were subsequently evaluated through sensitivity and uncertainty analyses to assess the robustness of the results. Infrastructure impacts were amortized over the system lifetime, transportation distances were based on national averages, and land-use change impacts were excluded. Methane flaring efficiencies were fixed where applicable; and accidental releases, bypass events, and downstream nutrient fate were not modeled.

The LCCA was conducted under the assumptions that energy, chemical, and labor prices remained constant over the analysis period, no carbon pricing or financing structures (e.g., loans or subsidies) were applied, and zero salvage value was realized at the end of the system lifetime. For the scenario involving co-products (algal biomass harvesting), it was further assumed that market prices remained stable, and no additional processing or transportation costs were incurred beyond those explicitly modeled.

As with most life-cycle assessments, the results are constrained by the system scale and flow assumptions used to construct the life cycle inventory. Three scenarios were based on real case study systems with design flows of 0.04, 0.2, and 0.4 MGD (AMB, CF and AM, respectively) while the remaining six were modeled using a representative flow of 1.0 MGD. Although results were normalized to a functional unit of 1 m^3^ of treated wastewater, infrastructure sizing and operational assumptions reflect these design capacities. Consequently, the findings may not fully represent system behavior or environmental impacts when extrapolated to substantially smaller or larger systems. Flow-rate sensitivity analysis was intended to assess moderate uncertainty around the assumed design conditions and did not capture the full range of flows possible in very small or very large wastewater treatment systems.

## 3. Results and discussion

### 3.1. Life cycle carbon footprint

**Fig. 2.**
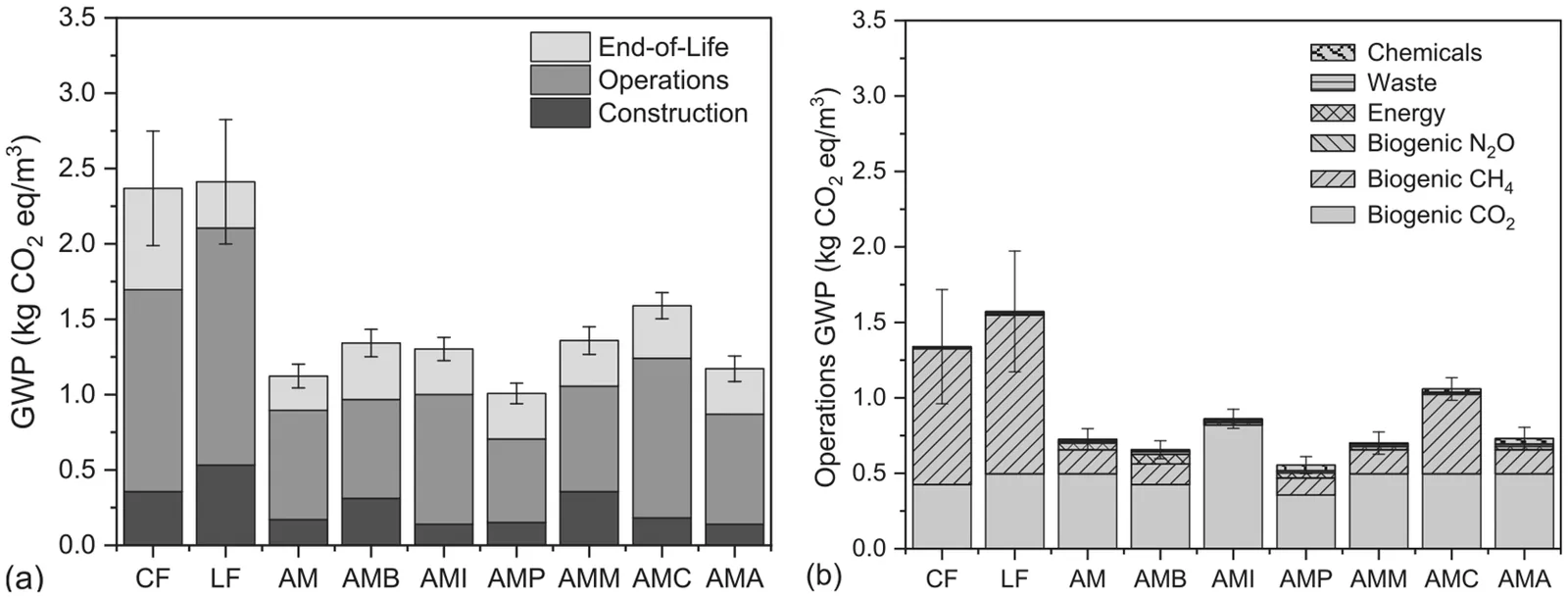
a. Life cycle carbon footprint of 9 lagoon alternatives. b: Operations carbon footprint. CF – clay-lined facultative; LF – plastic-lined facultative; AM – aerated lagoon + maturation pond; AMB - AM + biodomes; AMI – AM + iron-dosed UASB; AMP – AM + packed-bed reactor; AMM – AM + moving-bed-biofilm reactor; AMC – AM + constructed wetland; and AMA – AM + algal system. Error bars represent ±1 standard deviation from Monte Carlo uncertainty analysis.

The life cycle carbon footprint (measured as GWP) varied substantially across the nine treatment alternatives, with total emissions ranging from 1.01 to 2.41 kg CO_2_ eq/m^3^ of treated wastewater. This variation reflected differences in energy usage, direct and indirect emissions, infrastructure complexity, and end-of-life handling across scenarios. The baseline clay-lined facultative (CF) and HDPE-lined facultative (LF) systems exhibited the highest GWP values, at 2.37 and 2.41 kg CO_2_ eq/m^3^, respectively (Fig. 2a). The slightly higher footprint of LF stemmed from The slightly higher footprint of LF stemmed from its substantially higher design flow (1 MGD versus 0.2 MGD for CF), which resulted in greater organic loading and associated biogenic CH_4_ generation, as well as higher embodied carbon from HDPE liner production and installation. Similar embodied carbon contributions from liners have also been noted in previous studies (Morelli et al., 2018).

In contrast, aerated lagoon-maturation pond (AM) configurations with treatment enhancements exhibited lower total GWP. The standalone AM system reported a GWP of 1.12 kg CO_2_ eq/m^3^, with operations accounting for 54%, followed by end-of-life (26%) and construction (19%). Aeration led to a reduction in methane, thereby lowering the carbon footprint, consistent with previous findings (Vagheei, 2021). Other AM-based alternatives exhibited more distributed emissions across life cycle stages. For instance, AMB, which contained biofilm media in biodomes, recorded a GWP of 1.34 kg CO_2_ eq/m^3^, with 28% from construction.

Within operations, a key contributing phase, emissions were largely driven by biogenic sources (Fig. 2b). AMI, which integrated an iron-dosed upflow anaerobic sludge blanket reactor, showed a GWP of 0.82 kg CO_2_ eq/m^3^. This included flared methane, a mitigation strategy that converts methane into less harmful CO_2_. CO_2_ from flaring was treated as biogenic, consistent with IPCC definitions of emissions from non-fossil, biodegradable organics (US EPA, 2014). AMP, which included an aerated packed-bed biofilm reactor with woodchips, showed a comparable GWP of 0.81 kg CO_2_ eq/m^3^, though with slightly higher emissions from chemical inputs (Fig. 2).

AMC, which incorporated a constructed wetland, showed a GWP of 0.97 kg CO_2_ eq/m^3^, with operations contributing 45% and end-of-life 36%. Methane emissions from constructed wetlands typically result from anaerobic decomposition of organic matter in low-oxygen, high-loading zones. However, if fed with nitrified effluent wetlands are more likely to remain anoxic, thereby inhibiting methane generation (Abou-Elela, 2019). Decommissioning waste, including spent gravel, accumulated sludge, and vegetation, also added to the carbon footprint. However, this estimate did not account for carbon sequestered in vegetation. Notably, indirect emissions from chemicals (in AMP and AMA) were relatively low, at approximately 0.038 kg CO_2_ eq/m^3^.

Compared to previous studies, our results showed slightly higher carbon footprints for some scenarios. For example, systems coupled with constructed wetlands and algal treatment had footprints of 0.97 and 0.61 kg CO_2_ eq/m^3^, respectively, versus 0.69 and 0.57 kg CO_2_ eq/m^3^ reported by Garfí et al. (2017). This difference was likely due to the system boundary selection. While prior studies focused on standalone units, our analysis assessed treatment trains combined with an aerated lagoon, which introduced additional components and energy demands.

Furthermore, previous work by (Prateep Na Talang et al., 2022) suggested that electricity use for aeration and nutrient removal would dominate carbon footprints, yet our findings indicated that direct process emissions were the primary contributors. Our findings align with previous studies on the environmental impacts of small wastewater systems, which similarly identified direct greenhouse gas emissions as the dominant contributor to climate impacts, exceeding the contribution from electricity-related emissions (Awad et al., 2019). Importantly, while several studies as reviewed by Lam et al. (2020) excluded end-of-life stages, citing their negligible impact, our results showed that end-of-life processes contributed up to 37% of the total carbon footprint in some scenarios.

Taken together, these results demonstrate that while upgrades lowered operational emissions, construction and end-of-life impacts increased with complexity. Methane utilization would also be needed to realize full environmental benefits. Sustainable nutrient management will therefore require operational gains alongside attention to embodied carbon and end-of-life practices.

### 3.2. Life cycle eutrophication potential

#### 3.2.1. Freshwater eutrophication potential

Total freshwater eutrophication potential (FEP) ranged from 0.0039 kg P eq/m^3^ in AMP to 0.0153 kg P eq/m^3^ in CF. Across all scenarios, the operational phase was the primary contributor to freshwater eutrophication. CF had the highest FEP of all the scenarios (0.0153 kg P eq/m^3^), likely due to the low nutrient removal performance characteristic of standalone facultative lagoons, especially in cold climates. LF achieved only a modest 9% reduction compared to CF, reflecting limited improvement in phosphorus capture. Liner installation in LF was assumed to provide containment benefits and mitigated subsurface losses typically observed in the CF. This assumption was based on the greater variability and sensitivity of compacted clay liners to construction quality and long-term degradation compared to geomembrane liners installed under standard quality assurance and quality control procedures.

Enhanced AM-based configurations led to substantial reductions in FEP compared to the baseline. AMB achieved a 47% overall reduction while AMI reduced FEP by 39%. AMM resulted in a 23.6% reduction but was limited by higher construction-phase impacts. AMC (with a constructed wetland) and AMA achieved more modest FEP reductions of 7.2% and 18.3%, respectively, due to the limited phosphorus removal typically associated with these systems. Our FEP findings for AMC and AMA systems were an order of magnitude higher than those reported by Garfí et al. (2017) for FEP of standalone constructed wetlands and algal systems as independent unit processes. However, our analysis considered a full treatment train composed of several unit processes, making direct comparison limited.

In the operations phase, FEP was primarily driven by phosphorus emissions with negligible influence from energy, waste, and chemical inputs (Fig. 4b). CF and LF had the highest FEP (0.012 kg P eq/m^3^), mainly due to high phosphorus emissions. Poor removal of phosphorus is typical of facultative lagoons (von Sperling, 2007). AMC and AMA exhibited similar FEP levels, indicating limited improvement in phosphorus removal despite system changes. In contrast, AMP had 75% less FEP compared to CF and the lowest operational-phase emissions (0.002 kg P eq/m^3^) among all the alternatives. The observed phosphorus reduction resulted from biomass uptake, precipitation reactions, algal uptake, and sorption onto the woodchip medium, as reported in previous studies (Zhou et al., 2022). AMB's performance (FEP 0.006 kg P eq/m^3^) was attributed to algal uptake and incidental retention within the biofilm. For AMI (FEP 0.007 kg P eq/m^3^), phosphorus removal was through chemical precipitation of phosphorus as vivianite (Fe_3_(PO_4_)_2_·8H_2_O) under anaerobic conditions where Fe^2+^ reacted with phosphate (Yang et al., 2023).

**Fig. 3.**
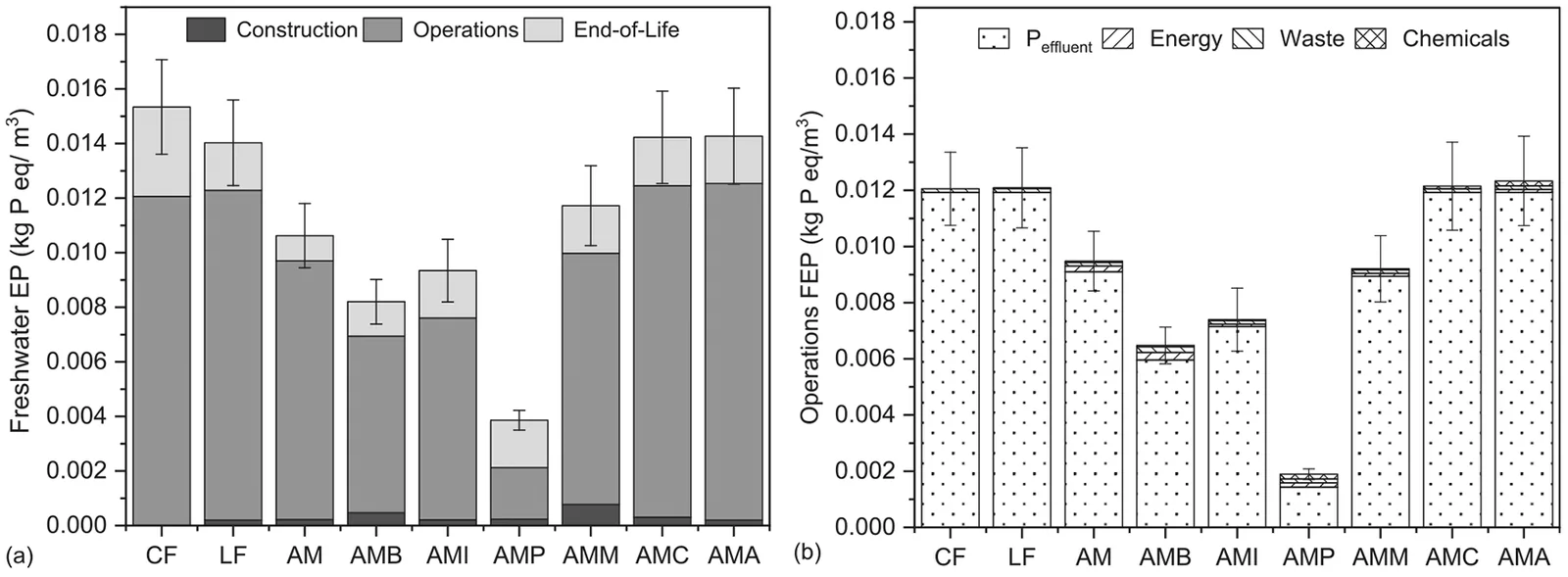
a. Life cycle freshwater eutrophication (FEP) in kg P eq/m^3^ for 9 alternative scenarios. b: Operations FEP. CF – clay-lined facultative; LF – HDPE-lined facultative; AM – aerated lagoon + maturation pond; AMB - AM + biodomes; AMI – AM + iron-dosed UASB; AMP – AM + packed-bed reactor; AMM – AM + moving-bed-biofilm reactor; AMC – AM + constructed wetland; and AMA – AM + algal system. Error bars represent ±1 standard deviation from Monte Carlo uncertainty analysis.

It is worth mentioning that the differences observed in Fig. 3a & b were driven primarily by scenario-specific effluent quality assumptions (Table S1) and operational inputs, particularly energy use and chemical dosing associated with nutrient removal processes. Differences in influent characteristics were minor across scenarios and had a comparatively small effect on the results, while construction-related inputs contributed little to the overall variation.

#### 3.2.2. Marine eutrophication potential (MEP)

Marine eutrophication potential (MEP), driven by nitrogen discharges, showed improvements in advanced scenarios. CF had the highest total MEP (0.009 kg N eq/m^3^) and LF offered minimal improvement, reducing total MEP by only 4%. AM-based systems were far more effective. AM reduced MEP by 18%, while AMB, AMP, and AMC achieved reductions between 48% and 54% compared to the baseline CF. The most notable performance was achieved by AMI and AMM, with MEP decreases of 75% and 72%, respectively, compared to the baseline. End-of-life contributions remained consistent across all systems (~0.002–0.003 kg N eq/m^3^), with modest variation depending on waste generated. Notably, AMI had a relatively high end-of-life contribution, attributed to desludging and the disposal of spent iron filings, which may retain nitrogen (Cao et al., 2016).

**Fig. 4.**
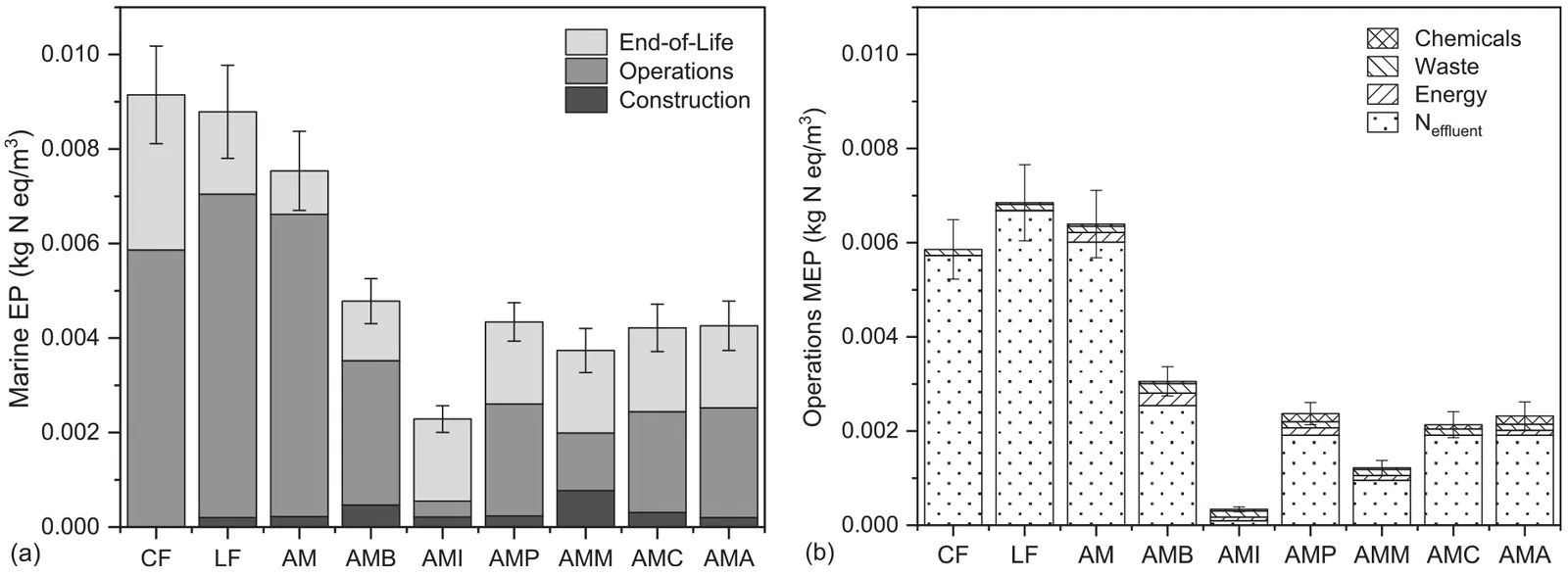
a. Life cycle marine eutrophication potential (MEP) in kg N eq/m^3^ for 9 nutrient management scenarios. b: Operations MEP. CF – clay-lined facultative; LF – plastic-lined facultative; AM – aerated lagoon + maturation pond; AMB - AM + biodomes; AMI – AM + iron-dosed UASB; AMP – AM + packed-bed reactor; AMM – AM + moving-bed-biofilm reactor; AMC – AM + constructed wetland; and AMA – AM + algal system. Error bars represent ±1 standard deviation from Monte Carlo uncertainty analysis.

The operations phase was a major contributor to MEP, primarily driven by direct nitrogen emissions from treatment processes rather than from energy, waste, or chemical inputs (Fig. 4b). CF set the baseline impact at 0.0057 kg N eq/m^3^. The most significant reductions in MEP were observed in AMI, resulting in a remarkable 98% decrease compared to CF. AMI's exceptional performance stemmed from biological denitrification facilitated by bacteria supported within the iron-dosed filter, which served as a growth medium (Lin et al., 2023). AMM reached a 59% reduction compared to the baseline, likely due to improved nitrogen removal through coupled nitrification-denitrification processes with the biofilm (Bhattacharya and Mazumder, 2021). AMB also performed well (0.0025 kg N eq), offering a balance between moderate emissions and operational performance.

When comparing our findings to previous studies, a key limitation was that most existing LCAs focused on standalone technologies, whereas our analysis considered integrated systems, where upstream and downstream processes significantly influenced overall impacts. For example, our MEP results for AMM (0.0037 kg N eq/m^3^) aligned with the 0.002 kg N eq/m^3^ reported by Awad et al. (2024) for a standalone MBBR system. The slightly higher impact in our case likely stemmed from upstream emissions included in our coupled system, which are typically excluded in standalone assessments (Piao et al., 2016). For algal systems, Kohlheb et al. (2020) reported 0.0002 kg N eq/m^3^ in a standalone setup, while our coupled configuration yielded 0.0023 kg N eq/m^3^. However, this was still within the range reported by Arashiro et al. (2018) for MEP of 0.002–0.016 kg N eq/m^3^. Similarly, Garfí et al. (2017) reported an MEP of 0.00014 kg N eq/m^3^ for a standalone constructed wetland, compared to our 0.0017 kg N eq/m^3^ when coupled to a lagoon system, although wetlands coupled to nitrifying lagoons are notably effective for nitrogen removal through denitrification (Almuktar et al., 2018). Ultimately, our results reinforced findings by Pausta et al. (2024) and Piao et al. (2016), that identified operational activities as key contributors to eutrophication potential.

In summary, for both freshwater and marine contexts, operational nutrient emissions were the primary driver of eutrophication potential, underscoring the importance of optimizing nutrient capture during active treatment. AMP and AMI emerged as the most effective alternatives, across both freshwater and marine systems, offering >70% reductions in eutrophication potential compared to the baseline (CF). While construction and end-of-life stages contributed modestly, our findings reinforce the need for targeted phosphorus and nitrogen management strategies, tailored to the receiving environment, to effectively mitigate eutrophication from lagoon treatment systems.

### 3.3. Life cycle cost analysis

**Fig. 5.**
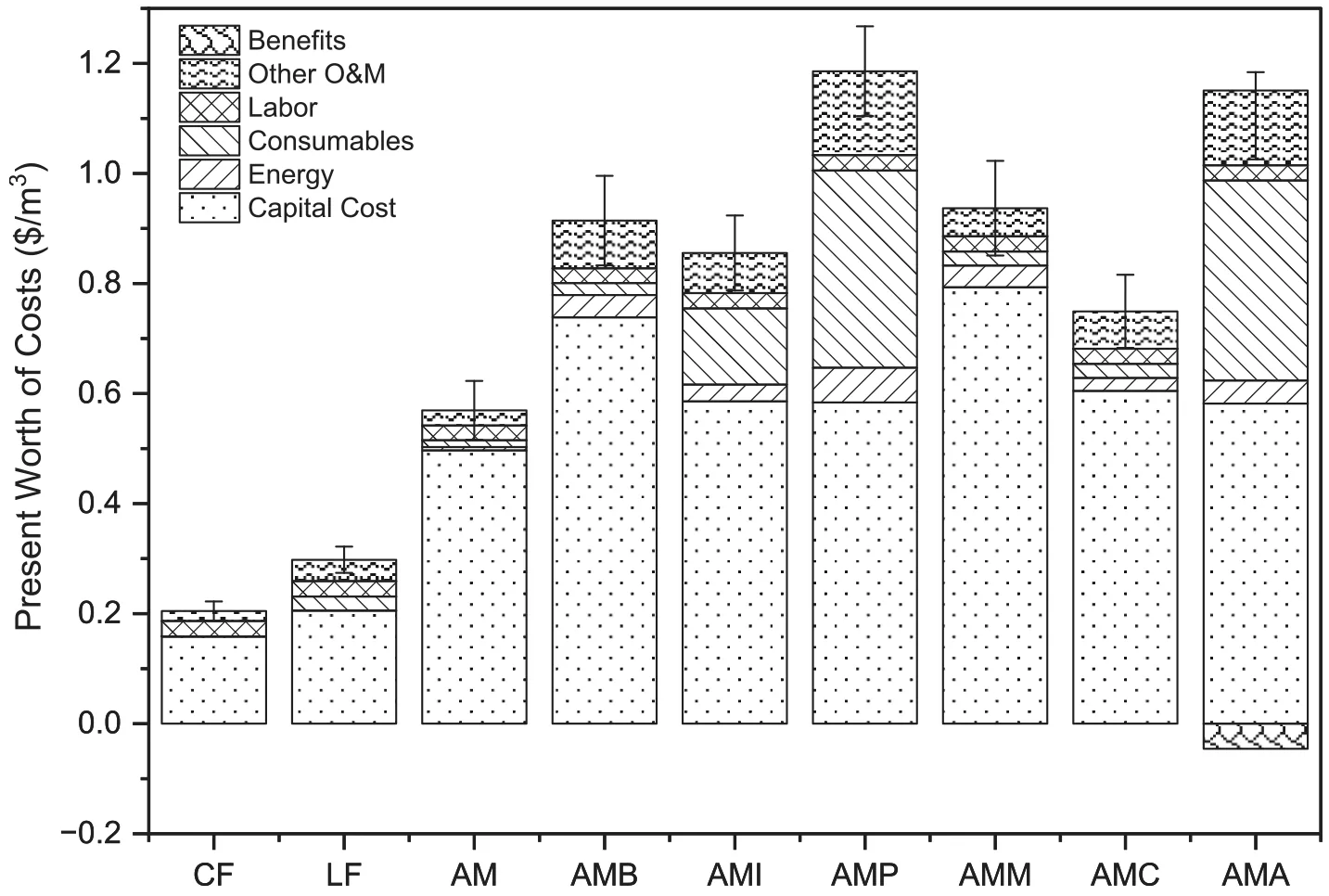
Present Worth of Costs per cubic meter of treated wastewater for each treatment scenario. CF – clay-lined facultative; LF – HDPE-lined facultative; AM – aerated lagoon + maturation pond; AMB - AM + biodomes; AMI – AM + iron-dosed UASB; AMP – AM + packed-bed reactor; AMM – AM + moving-bed-biofilm reactor; AMC – AM + constructed wetland; and AMA – AM + algal system. Error bars represent ±1 standard deviation from Monte Carlo uncertainty analysis.

The Present Worth of Cost (PWC) reflected the total life cycle cost of each treatment scenario, evaluated over a 20-year analysis period at a 6% interest rate (Fig. 5). The baseline (CF) presented the lowest PWC at $0.20/m^3^, primarily due to its low capital cost of $0.16/m^3^ and negligible recurring operational inputs. A slight increase was observed in the LF scenario, which rose to $0.30/m^3^. This increase was largely attributed to a modest rise in capital costs (liner installation), although the system still lacked significant process enhancements.

As the treatment systems become more advanced, the PWC rose considerably. The AM scenario saw a more than twofold increase in cost to $0.57/m^3^, driven largely by a higher capital investment of $0.50/m^3^, attributed to aeration and the consequent operational inputs (energy), albeit relatively low. In contrast, the AMB and AMI scenarios had high costs ($0.91, and $0.86/m^3^ respectively). AMB incurred higher capital, energy, and O&M expenses, which were attributed to the installation of biodomes and continuous aeration in addition to the already existing aeration in the lagoon upstream. Notably, AMI had elevated consumables cost ($0.14/m^3^), attributed to chemicals and iron fillings.

AMP stood out with the highest PWC of $1.19/m^3^. Although capital costs were moderate ($0.58/m^3^), there were substantial chemical ($0.36/m^3^) and other O&M costs such as preventative maintenance ($0.15/m^3^). AMM ranked among the more expensive options at $0.94/m^3^, largely due to high capital cost for setting up the MBBR unit and moderate operational costs mainly from energy and consumables. AMA, though similar to AMP in terms of consumables and O&M costs, earned revenue from harvested algal biomass ($0.05/m^3^), reducing PWC down to $1.10/m^3^. AMC emerged as a relatively cost-effective advanced scenario, with a total PWC of $0.75/m^3^. It is important to note that these per-m^3^ costs reflected treatment only and did not include collection system costs, monitoring, compliance, billing, or administrative overhead, which can significantly increase community-level service charges.

Cost comparisons with previous studies were complicated by inconsistent reporting, as several studies report only capital and operational costs without accounting for present value or focus solely on standalone systems rather than coupled configurations (Arashiro et al., 2018; Garfí et al., 2017). In our analysis, capital costs ($0.20–$1.19/m^3^) were lower than the $1.90–$2.60/m^3^ reported by Morelli et al. (2018) likely due to our narrower system boundary, which excluded the collection costs included in their estimate. Our findings aligned with Bertanza et al. (2017) who reported $0.65–$1.30/m^3^ for nutrient management upgrades. Similarly, our capital and O&M estimates were comparable to those reported for constructed wetlands and algal systems by Garfí et al. (2017) and Arashiro et al. (2018), though those were based on standalone technologies. For MBBR systems, Awad et al. (2024) reported ~$0.56/m^3^, while our coupled system was higher at $0.90/m^3^, reflecting the added complexity of integration. Overall, our operational cost trends were consistent with those reported for integrated systems by Piao et al. (2016), highlighting the influence of system boundaries and configuration on total cost outcomes.

### 3.4. Scenario selection and trade-offs

**Fig. 6.**
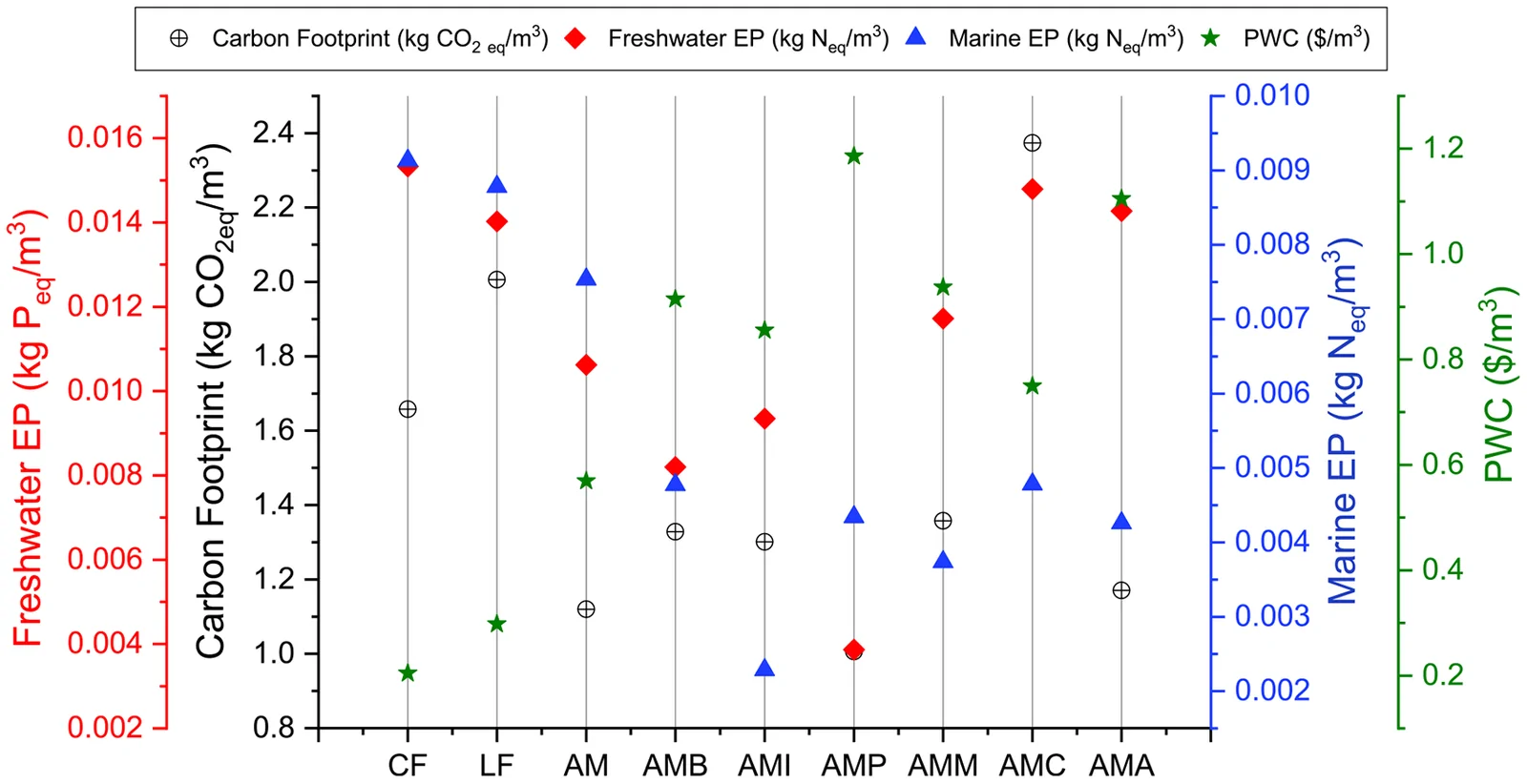
Combined environmental (carbon footprint, FEP, MEP) and cost (PWC) analysis for 9 alternative scenarios. Lower values mean better performance. CF – clay-lined facultative; LF – plastic-lined facultative; AM – aerated lagoon + maturation pond; AMB - AM + biodomes; AMI – AM + iron-dosed UASB; AMP – AM + packed-bed reactor; AMM – AM + moving-bed-biofilm reactor; AMC – AM + constructed wetland; and AMA – AM + algal system.

**Fig. 7.**
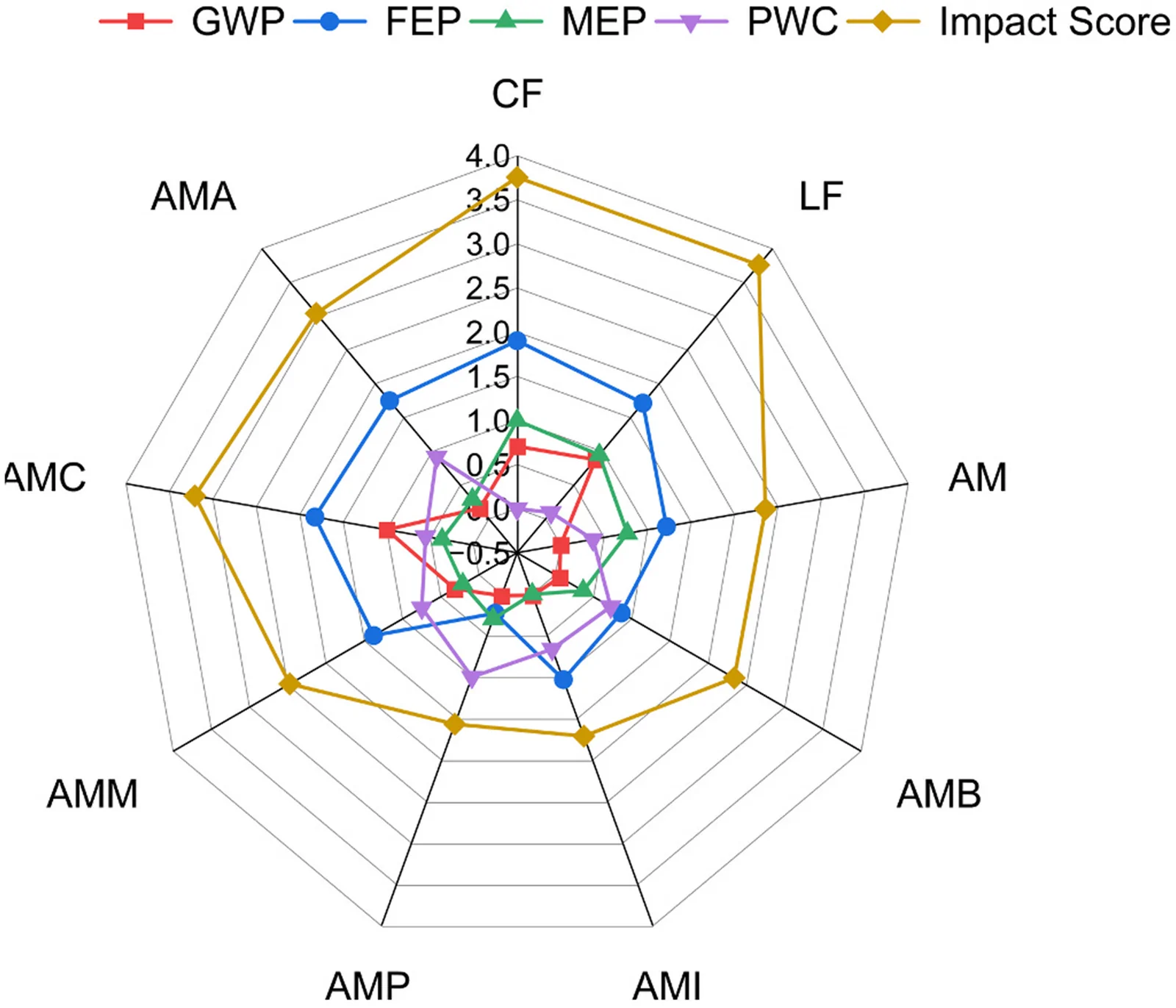
Radar plot showing the multi-criteria scoring of nine nutrient management alternatives. The scale represents the unweighted sum of normalized, dimensionless values derived from min–max scaling of each indicator (GWP: global warming potential; FEP: freshwater eutrophication potential; MEP: marine eutrophication potential; and PWC: present worth of cost). Lower composite scores indicate better overall environmental–economic performance; therefore, scenarios plotted closer to the center represent more favorable overall performance. CF – clay-lined facultative; LF – HDPE-lined facultative; AM – aerated lagoon + maturation pond; AMB – AM + biodomes; AMI – AM + iron-dosed UASB; AMP – AM + packed-bed reactor; AMM – AM + moving-bed biofilm reactor; AMC – AM + constructed wetland; and AMA – AM + algal system.

The comparative evaluation of treatment scenarios revealed key trade-offs between environmental performance and cost. Using impact values for GWP, FEP, MEP, and PWC, distinct patterns emerged (Figs. 6 and 7). As shown on Fig. 6, the baseline (CF), though the most cost-effective at $0.20/m^3^, performed poorly, particularly in FEP (0.015 kg P eq/m^3^, the highest) and MEP (0.009 kg N eq/m^3^), ranking 8th overall. LF, a marginally improved variant, showed slightly lower eutrophication levels but increased carbon emissions (2.41 kg CO_2_ eq/m^3^) and cost ($0.30/m^3^), resulting in a tied overall score with CF but ranked last (9th) due to higher environmental burden. However, although CF performed poorly in this study, facultative lagoons can be effective for nutrient removal in warm climates (von Sperling, 2007).

AMP emerged as the top-ranked scenario based on a single impact score, despite being the most expensive ($1.19/m^3^). AMP had the lowest freshwater eutrophication potential (0.00386 kg P eq/m^3^) and one of the lowest MEP values (0.004 kg N eq/m^3^), reflecting the effectiveness of its nutrient removal strategy. AMPs moderate GWP (1.007 kg CO_2_ eq/m^3^) and the lowest overall normalized performance earned it the highest rank (1st) with a score of 1.53.

AMI ranked 2nd overall, combining high MEP performance (0.002 kg N eq/m^3^, the lowest) with low GWP (1.30 kg CO_2_ eq/m^3^) and moderate cost ($0.86/m^3^). The AMI impact score of 1.90 indicated strong environmental benefits at a somewhat more accessible cost than AMP. However, it is important to note that AMI was modeled based of a lab-scale technology, and its sustainability at full scale is still uncertain compared to field-tested systems.

Using the radar plot (Fig. 7), clear performance differences emerged among the evaluated treatment alternatives when environmental and economic criteria were considered simultaneously. Scenarios incorporating advanced nutrient management downstream of aerated lagoons, specifically AMP, AMI, and AMB, clustered closest to the center of the plot, indicating the lowest composite scores and the most favorable overall performance. These configurations therefore represented the most suitable options based on the combined assessment of climate impacts, eutrophication control, and cost. In contrast, scenarios without dedicated nutrient removal or with less efficient treatment configurations plotted farther from the center, reflecting higher composite scores and less favorable trade-offs between environmental benefits and economic feasibility.

This pattern arose because advanced nutrient management technologies substantially reduced nitrogen and phosphorus loads in the effluent, thereby lowering freshwater and marine eutrophication impacts, while aerated lagoon operation improved organic matter removal and reduced methane generation relative to conventional facultative systems. Although these configurations required higher energy and capital inputs, the environmental gains, particularly in eutrophication control and greenhouse gas mitigation, outweighed the additional costs when evaluated across all criteria, resulting in lower composite scores. Detailed scenario-specific impact and cost values and individual rankings are provided in the Supplementary Information.

A key limitation of this ranking method was its equal weighting of all four metrics, which may have overemphasized environmental performance at the expense of cost. This is particularly important for small communities, where affordability often outweighs environmental benefits. As a result, high-cost systems may have ranked higher than what is practical or locally appropriate. To address this, future studies could apply a multi-criteria decision analysis (MCDA) approach, enabling users to assign weights based on local priorities and better balance trade-offs among cost, carbon, and eutrophication impacts.

### 3.5. Sensitivity and uncertainty analysis

A sensitivity analysis showed that for carbon footprint, the highest sensitivity factors were associated with design flow, liner materials, waste sludge, and waste plastics. The sensitivity of several scenarios to design flow highlights the importance of infiltration and inflow management in small-community lagoon systems. Uncontrolled increases in flow can elevate hydraulic and organic loading and increase operational costs. For eutrophication potential, effluent nutrient concentrations had the highest sensitivity factors. Inputs with high sensitivity factors were considered more influential, indicating that better-quality data and site-specific estimates are essential for reliable life cycle modeling and decision-making. Capital cost, analysis period, and design flow were identified as the most critical factors influencing lifetime cost estimates, highlighting that long-term financial planning hinges on accurate infrastructure cost estimation and realistic flow projections. Notably, for systems serving populations of fewer than 1000 people, unit costs are expected to be considerably higher due to reduced economies of scale and the disproportionate impact of capital, O&M, and compliance costs relative to flow.

Monte Carlo simulations revealed some uncertainty in environmental impacts, with GWP consistently showing low standard error of mean indicating high precision in the data used, although true uncertainty may be broader in practice due to variability in published emissions data. MEP and FEP showed similar trends, reinforcing the reliability of environmental performance estimates across treatment alternatives. Economic uncertainty analysis showed that the highest uncertainty occurred in more complex systems such as AMP, primarily due to scale-dependent capital costs. The narrow standard errors and confidence intervals underscored the robustness of the estimates and overall confidence in the LCA and LCCA results (Salciccioli et al., 2016). Detailed results tables are provided in Supplementary Tables 4 and 7.

### 3.6. Limitations and future work

As with any modeling-based analysis, several limitations should be acknowledged when interpreting the results and applying them to site-specific decision making. Factors such as operator expertise, local construction practices, land availability, community acceptance, and institutional capacity were not explicitly modeled, although they may influence technology feasibility and long-term performance. While these factors are unlikely to change the overall direction of the results, they may affect the relative attractiveness of specific technologies for individual communities.

The analysis was conducted for systems within the flow range typical of small communities and should be interpreted accordingly. Extrapolation to substantially smaller decentralized systems or larger municipal facilities may alter both environmental and economic performance due to scale effects, infrastructure sizing, and operational complexity. In addition, the environmental results reflect current electricity grid conditions. As regional and national grids decarbonize, the contribution of electricity consumption to overall greenhouse gas emissions is expected to decline, which may reduce the relative advantage of lower-energy systems and increase the importance of direct process emissions such as methane and nitrous oxide. Under a low-carbon grid scenario, treatment configurations with higher electricity demand but strong nutrient removal performance may become comparatively more favorable.

The study assumed a 20-year system lifetime, consistent with common practice in comparative LCCA studies; however, lagoon systems often operate for 30–40 years or longer. Extending the design life would increase the relative importance of operational impacts and costs compared to construction, potentially favoring technologies with lower long-term energy and chemical requirements. Preliminary sensitivity testing indicated that extending the analysis period would change absolute costs and impacts but would be unlikely to substantially alter the relative ranking of scenarios; nevertheless, future work should explicitly evaluate longer design horizons.

Site-specific conditions such as climate, influent characteristics, sludge management practices, and hydraulic constraints were not explicitly represented and may favor certain technologies over others. Previous lagoon LCA studies have shown considerable variability among constructed systems of the same technology, underscoring the importance of local conditions in shaping environmental performance. In addition, variability in state and local design regulations may impose requirements that differ from those assumed here, potentially affecting liner specifications, effluent limits, redundancy requirements, or monitoring intensity. Users of these results should therefore exercise caution when applying them directly to regulatory contexts that deviate from the design assumptions used in this study.

Finally, the cost estimates were derived from design-based modeling, literature sources, and vendor information and should be interpreted as planning-level estimates consistent with AACE Class 3 accuracy (approximately ±10–30%). Future research should prioritize validation using empirical cost data from implemented lagoon upgrade projects, including data available through state revolving loan fund programs and utility records. Expanding the framework to incorporate social indicators, community risk tolerance, and implementation barriers would further strengthen its value as a decision-support tool for small and rural wastewater utilities.

## 4. Conclusion

This study evaluated nine nutrient management scenarios for small community lagoon wastewater treatment systems using an integrated life cycle assessment and life cycle cost framework. The results revealed clear trade-offs between environmental performance and economic feasibility across treatment configurations. Baseline systems such as CF and LF were the most affordable but consistently exhibited the highest impacts, particularly for eutrophication and greenhouse gas emissions. In contrast, advanced configurations including AMP and AMI achieved the largest reductions in nutrient pollution and carbon footprint, exceeding 70% in some cases, at higher cost. Intermediate options such as AMB and AM provided a balanced compromise between environmental improvement and economic feasibility, representing practical upgrade pathways for communities seeking enhanced treatment without prohibitive investment.

The composite scoring and multi-criteria analysis further demonstrated that treatment configurations coupling aerated lagoons with targeted nutrient removal technologies, particularly packed-bed reactors, iron-dosed UASB systems, and biodomes, offered the most favorable overall performance when climate impacts, eutrophication control, and cost were considered simultaneously. These findings reinforce the importance of evaluating wastewater treatment technologies using multiple sustainability metrics rather than cost alone.

By integrating environmental performance, economic analysis, sensitivity testing, and uncertainty assessment, this study provides a robust framework for comparing lagoon upgrade options beyond upfront capital costs alone. The results offer practical insights for utilities, engineers, and regulators seeking to identify sustainable treatment pathways for small and rural communities and support more informed decision making regarding long-term wastewater infrastructure investments.

## Supplementary Material

Supplementary tables

Supplementary material

## Figures and Tables

**Table 1:** Summary of life cycle inventory items across construction, operation, and end-of-life stages for each scenario.

Life cycle stage	Inventory item	Unit
Construction	Excavation & earthworks diesel	MJ m^−3^
	Concrete (headworks, basins, reactors)	m^3^ m^−3^
	Reinforcement steel	kg m^−3^
	Liners (clay/HDPE)	kg m^−3^
	Plastic media (biofilm carriers, floating covers)	kg m^−3^
	Baffles	kg m^−3^
	Aeration infrastructure (piping)	kg m^−3^
Operation	Electricity use (treatment + nutrient removal)	kWh m^−3^
	Diesel use (O&M, solids handling)	MJ m^−3^
	Influent & effluent BOD_5_, TN, TP	mg L^−1^
	Air emissions (CH_4_, CO_2_, N_2_O)	kg CO_2_-eq m^−3^
	Water emissions (N&P)	kg N/P-eq m^−3^
	Sludge production	kg m^−3^
	Screenings and grit disposal	kg m^−3^
	Chemical use (disinfection, nutrients, pH)	kg m^−3^
End-of-life	Desludging	kg m^−3^
	Solids transport	ton-km m^−3^
	Decommissioning diesel	MJ m^−3^

**Table 2:** Data quality assessment of major datasets used in the LCA and LCCA. Overall quality was assigned based on the least pedigree score of the data quality index (DQI): 1-high; 2-good; 3-medium; 4-low and 5-very low.

Dataset	Reliability	Temporal	Geographic	Technological	Completeness	DQI	Quality
LCA Inventory Datasets							
Construction (excavation, concrete, steel, liners, media, piping)	2	2	2	1	1	(2,2,2,1,1)	Good
Electricity consumption	1	1	1	1	1	(1,1,1,1,1)	High
Diesel consumption	2	2	1	1	1	(2,2,1,1,1)	High
Influent and effluent water quality	1	1	1	1	1	(1,1,1,1,1)	High
Air emissions (CH_4_, CO_2_, N_2_O)	3	2	3	3	2	(3,2,3,3,2)	Medium
Water emissions (TN, TP)	2	1	1	1	1	(2,1,1,1,1)	High
Sludge production and management	2	2	2	1	2	(2,2,2,1,2)	Good
Chemical consumption	2	2	2	2	1	(2,2,2,2,1)	Good
Transportation (solids hauling)	2	2	1	1	1	(2,2,1,1,1)	High
LCCA Datasets							
Site-specific operating costs (labor, electricity, chemicals, equipment)	1	1	1	1	1	(1,1,1,1,1)	High
Vendor quotes (capital costs)	2	2	2	1	1	(2,2,2,1,1)	Good
RSMeans construction cost data	2	2	2	2	1	(2,2,2,2,1)	Good
Solids handling costs	2	2	1	1	2	(2,2,1,1,2)	Good
Resource recovery revenues	3	2	3	2	2	(3,2,3,2,2)	Medium

## Data Availability

Data will be made available on request.

## HIGHLIGHTS

- Environmental and cost analysis of nine nutrient management strategies in lagoons.
- Biogenic emissions from operations contributed most to environmental impacts.
- Low-cost upgrades (<$0.30/m^3^) while affordable, cut eutrophication <25%.
- Advanced options cut eutrophication >70%, though requiring higher overall costs.

## Appendix A. Supplementary data

Supplementary data to this article can be found online at https://doi.org/10.1016/j.scitotenv.2026.181978.
