# Supplementary material for: Life cycle environmental and economic impacts of nutrient management in small community lagoon wastewater systems
Source: Sci Total Environ. Author manuscript; Available in PMC 2026 Jul 29. (PMC13418470; doi:10.1016/j.scitotenv.2026.181978)
Supplement: Supplementary material [file NIHMS2194708-supplement-Supplementary_material.docx]

**Life Cycle Environmental and Economic Impacts of**

**Nutrient Management in Small Community Lagoon Wastewater Systems**

Denis Ruto, Corbin Vincent, Ziya Jang, Pablo Cornejo, Harold Leverenz,

Steffen Mehl, and Kevin Orner

**Supplementary Information**

# Scenario Selection

Following a previous comprehensive review by the authors of 1,003 published studies on nutrient management strategies applicable to lagoon systems, the most prevalent and practically implementable solutions were selected as possible scenarios. The scenarios were set up in a way that they incrementally progressed in upgrades and performance, starting from the most basic treatment configuration still in use. The remaining scenarios reflect a progressive sequence of upgrades, starting with low-cost enhancements such as installing a high-density polyethylene liner, and introducing aeration, followed by increasingly complex interventions. These included enhanced biological systems (biodomes: submerged, dome-shaped biofilm reactors, submerged, aerated packed-bed biofilm reactor, and moving-bed biofilm reactor), nature-based systems (constructed wetland), nutrient recovery through phosphorus precipitation as vivianite (iron-dosed upflow anaerobic sludge blanket (UASB)), and resource recovery from algae cultivation (high-rate algal pond). Although development of iron-dosed UASB is at a bench-scale stage, it was included as a scenario owing to the promising performance and benefit of nutrient recovery. Three of the nine scenarios (CF, AM, and AMB) were based on actual case studies, while the performance data for the remaining six scenarios were drawn from the literature under assumed temperate climate conditions.

# Life Cycle Impact Assessment and Interpretation

To support environmental impact evaluation, influent and effluent quality values used for each scenario are provided in Table S1.

Table S1: Influent and effluent quality data of nine scenarios

| **Influent quality** | **CF** | **LF** | **AM** | **AMB** | **AMI** | **AMP** | **AMM** | **AMC** | **AMA** |
| --- | --- | --- | --- | --- | --- | --- | --- | --- | --- |
| Flow Rate (m^3^/day) | 757.08 | 3,785.40 | 1,514.16 | 151.42 | 3,785.40 | 3,785.40 | 3,785.40 | 3,785.40 | 3,785.40 |
| BOD_5_ (mg/L) | 300.00 | 350.00 | 350.00 | 300.00 | 300.00 | 250.00 | 350.00 | 350.00 | 350.00 |
| TN (mg/L) | 80.00 | 60.00 | 42.00 | 23.20 | 20.00 | 60.00 | 60.00 | 60.00 | 60.00 |
| TP (mg/L) | 8.00 | 15.00 | 8.50 | 8.00 | 10.00 | 15.00 | 15.00 | 15.00 | 15.00 |
| NH_3_ (mg/L) | 37.00 | 45.00 | 36.70 | 35.00 | 7.00 | 45.00 | 45.00 | 45.00 | 45.00 |
| pH | 8.00 | 7.00 | 7.00 | 7.00 | 7.00 | 7.00 | 7.00 | 7.00 | 7.00 |
| **Effluent quality** |  |  |  |  |  |  |  |  |  |
| Flow Rate (m^3^/day) | NA | 3,785.40 | 1,514.16 | 151.42 | 3,785.40 | 3,785.40 | 3,785.40 | 3,785.40 | 3,785.40 |
| BOD_5_ (mg/L) | 57.00 | 30.00 | 30.00 | 46.00 | 15.00 | 20.00 | 30.00 | 30.00 | 30.00 |
| TN (mg/L) | 30.00 | 35.00 | 31.50 | 13.30 | 0.50 | 10.00 | 5.00 | 10.00 | 10.00 |
| TP (mg/L) | 5.00 | 5.00 | 3.82 | 2.50 | 3.00 | 0.60 | 3.75 | 5.00 | 5.00 |
| NH_3_ (mg/L) | 0.50 | 22.50 | 15.00 | 0.90 | 0.00 | 0.60 | 1.00 | 2.00 | 1.00 |
| pH | 8.20 | 8.00 | 7.40 | 6.90 | 7.00 | 8.00 | 8.00 | 8.00 | 8.00 |

Notably, in practice, the effluent quality may vary considerably depending on lagoon size, loading rate, and temperature. In particular, CF, LF, AM, AMC, and AMA were highly sensitive to these factors. As a result, freshwater and marine eutrophication potentials (FEP and MEP) could differ for better-performing systems of the same type. This variability highlights the importance of context-specific operating conditions when interpreting scenario performance.

# Data Quality Assessment

## 3.1 Data Quality Goals

Data quality was assessed following the pedigree matrix approach of Weidema and Wesnæs (1996) and the U.S. EPA guidance for life-cycle inventory data quality assessment (Edelen and Ingwersen, 2016). Data quality goals were established for five key indicators: reliability, temporal, geographical, and technological representativeness, completeness, consistency, and uncertainty as shown in Table S2.

Table S2: Data quality goals used for evaluating inventory data in the LCA and LCCA (adapted from (Edelen & Ingwersen, 2016; Weidema & Wesnæs, 1996)

| **Data Quality Indicator** | **Data Quality Goal for This Study** |
| --- | --- |
| **Reliability** | Prioritize measured lagoon operational data, monitoring records, design reports, and peer-reviewed literature. Distinguish primary, secondary, and background datasets. |
| **Temporal Representativeness** | Use recent (2022 or later) data representative of current technologies and regulations. Prefer datasets covering ≥1 full year of operation and multi-year records where available to capture seasonal and climatic variability. |
| **Geographical Representativeness** | Prioritize U.S.-based data. State- or region-specific datasets preferred; national datasets used when regional data were unavailable (UN geoscheme Level D/E). |
| **Technological Representativeness** | Data should represent modeled technology with respect to process design, operating conditions, material characteristics, and process scale. |
| **Completeness** | Include all significant inputs and outputs within the system boundary, including materials, energy, infrastructure, transportation, nutrient flows, sludge management, and direct emissions to air, water, and soil. |

Reliability prioritized measured operational data, monitoring records, and peer-reviewed sources. Temporal representativeness favored recent datasets (2022 or later) reflecting current lagoon operating conditions, preferably covering at least one year of operation. Geographical representativeness prioritized U.S.-based and regional datasets, with national-level data used when state/regional information was unavailable. Technological representativeness was evaluated based on process design, operating conditions, material characteristics, and process scale. Completeness was assessed by verifying inclusion of all significant material, energy, nutrient, infrastructure, transportation, waste management, and emission flows within the system boundaries.

## 3.2 Pedigree Matrix Criteria

Data quality was evaluated using the pedigree matrix criteria shown in Table S3, adapted from Weidema and Wesnæs (1996) and Edelen and Ingwersen (2016). Each criterion was scored on a scale of 1 (highest quality) to 5 (lowest quality).

Table S3: Pedigree matrix criteria used for data quality assessment of life-cycle inventory and cost datasets, adapted from Weidema and Wesnæs (1996) and Edelen and Ingwersen (2016). Scores range from 1 (highest data quality) to 5 (lowest data quality).

| **Indicator** | **1 (Highest Score)** | **2** | **3** | **4** | **5 (Lowest Score)** |
| --- | --- | --- | --- | --- | --- |
| **Reliability** | Verified data based on measurements | Verified data based on a calculation or non-verified data based on measurements | Non-verified data based on a calculation | Documented estimate | Undocumented estimate |
| **Temporal correlation** | Less than 3 years of difference | Less than 6 years of difference | Less than 10 years of difference | Less than 15 years of difference | Age of data unknown or more than 15 years |
| **Geographical correlation** | Data from same resolution and same area of study | Within one level of resolution and a related area of study | Within two levels of resolution and a related area of study | Outside of two levels of resolution but a related area of study | From a different or unknown area of study |
| **Technological correlation** | All technology categories are equivalent | Three of the technology categories are equivalent | Two of the technology categories are equivalent | One of the technology categories is equivalent | None of the technology categories are equivalent |
| **Completeness** | Representative data from >80% sample of sites over an adequate period | Representative data from 60–79% sample of sites over an adequate period, or >80% over a shorter period | Representative data from 40–59% sample of sites over an adequate period, or 60–79% over a shorter period | Representative data from <40% sample of sites over an adequate period, or 40–59% over a shorter period | Unknown, or data from a small number of sites and from shorter periods |

# Sensitivity Analysis

Sensitivity analysis was performed to evaluate how variations in key input parameters influenced the environmental and economic outcomes of each treatment scenario. Variables contributing less than 5% to overall impacts were excluded (Salciccioli et al., 2016). The remaining inputs were independently varied by ±20%, while all others were held constant (Jaczynska et al., 2025). Sensitivity factors were calculated as described below, over a spread of 40% to account for ±20% change.

$Sensitivity Factor =\frac{\Delta\mathrm{Output}}{\Delta\mathrm{Inputs}}$ = $\frac{\frac{{[\Delta}_{(+20\%)}-\Delta_{\left( -20\% \right)}]}{\mathrm{original}}}{0.4}$

(1)

Parameters with higher sensitivity factors are considered more influential, indicating that better-quality data and site-specific estimates are essential for reliable life cycle modeling and decision-making (Cornejo et al., 2013). Detailed results tables are provided in Supplementary *Tables 3 and 5.*

## 4.1 Environmental Sensitivity

Environmental impacts were assessed based on global warming potential (GWP) and eutrophication potential (EP), with the most influential inputs (>50% contribution) varying depending on the impact category and treatment scenario. Sensitivity analysis of EP was conducted using aggregated input parameters, treating FEP and MEP as a single category. For GWP, the most sensitive parameters were design flow, capital-related inputs (e.g., liner material), waste sludge, and waste plastics. Flow rate had a strong influence in advanced scenarios like AMM (0.29), AMB (0.28), and AMC (0.17), reflecting how changes in assumed flow volumes can significantly affect emissions per cubic meter of treated wastewater (Table S4). This is especially relevant for small or variable-flow systems.

Table S4: Sensitivity factors for Global Warming Potential. Red shades indicated highly sensitive inputs

| **Inputs Varied (+/-20%)** | **CF** | **LF** | **AM** | **AMB** | **AMI** | **AMP** | **AMM** | **AMC** | **AMA** |
| --- | --- | --- | --- | --- | --- | --- | --- | --- | --- |
| Excavation | 0.00 | 0.00 | 0.00 | 0.00 | 0.00 | 0.00 | 0.00 | 0.00 | 0.00 |
| Liner | 0.00 | 0.04 | 0.14 | 0.22 | 0.07 | 0.13 | 0.10 | 0.11 | 0.12 |
| Concrete | 0.00 | 0.00 | 0.02 | 0.01 | 0.06 | 0.01 | 0.00 | 0.00 | 0.00 |
| Flow rate | 0.00 | 0.05 | 0.19 | 0.28 | 0.05 | 0.16 | 0.29 | 0.17 | 0.14 |
| Biogenic CO2 | 0.00 | 0.00 | 0.00 | 0.00 | 0.00 | 0.00 | 0.00 | 0.00 | 0.00 |
| Effluent Nitrogen | 0.00 | 0.00 | 0.00 | 0.00 | 0.00 | 0.00 | 0.00 | 0.06 | 0.00 |
| Effluent Phosphorus | 0.00 | 0.00 | 0.00 | 0.00 | 0.00 | 0.00 | 0.00 | 0.00 | 0.00 |
| Treatment Energy | 0.00 | 0.00 | 0.05 | 0.05 | 0.02 | 0.03 | 0.02 | 0.06 | 0.02 |
| Waste Sludge | 0.12 | 0.05 | 0.08 | 0.05 | 0.16 | 0.16 | 0.00 | 0.14 | 0.14 |
| Waste Concrete | 0.00 | 0.00 | 0.00 | 0.00 | 0.00 | 0.00 | 0.00 | 0.00 | 0.00 |
| Waste Plastics | 0.00 | 0.05 | 0.16 | 0.28 | 0.15 | 0.15 | 0.00 | 0.00 | 0.14 |

Waste sludge handling showed a notable influence in scenarios like AMI and AMP (0.16 each), and AMA/AMC (0.14 each), highlighting the importance of accurately quantifying sludge volumes and disposal practices. Waste plastics also significantly impacted results in AMB (0.28) and AMP (0.15), underscoring the carbon impact of material inputs, especially in chemically intensive systems. In contrast, inputs such as excavation, biogenic CO₂, and concrete had negligible effects across all scenarios.

For EP, effluent nutrient concentrations were the dominant drivers (Table S5). Effluent phosphorus exhibited the highest sensitivity, particularly in AMI (0.81), AMM (0.70), AMC (0.69), and AMA (0.69). This reflects the critical role of nitrogen and phosphorus removal in minimizing both FEP and MEP, and the need for precise data on effluent discharge quality. Effluent nitrogen was similarly impactful, especially in AMP and AM (0.47 each) and LF (0.45). These findings make clear that even small inaccuracies in effluent quality estimates can lead to substantial uncertainty in impact assessment. Other inputs, including treatment energy and construction materials, were found to have minimal influence on eutrophication outcomes.

Table S5: Sensitivity factors for Eutrophication Potential. Dark red shades indicate highly sensitive inputs.

| **Inputs Varied (+/-20%)** | **CF** | **LF** | **AM** | **AMB** | **AMI** | **AMP** | **AMM** | **AMC** | **AMA** |
| --- | --- | --- | --- | --- | --- | --- | --- | --- | --- |
| Excavation | 0.00 | 0.00 | 0.00 | 0.00 | 0.00 | 0.00 | 0.00 | 0.00 | 0.00 |
| Liner | 0.00 | 0.00 | 0.00 | 0.01 | 0.01 | 0.01 | 0.00 | 0.00 | 0.00 |
| Concrete | 0.00 | 0.00 | 0.00 | 0.00 | 0.00 | 0.00 | 0.00 | 0.00 | 0.00 |
| Flow rate | 0.00 | 0.00 | 0.00 | 0.01 | 0.01 | 0.01 | 0.02 | 0.01 | 0.00 |
| Biogenic CO2 | 0.00 | 0.00 | 0.00 | 0.00 | 0.00 | 0.00 | 0.00 | 0.00 | 0.00 |
| Effluent Nitrogen | 0.33 | 0.45 | 0.47 | 0.22 | 0.02 | 0.47 | 0.13 | 0.19 | 0.19 |
| Effluent Phosphorus | 0.40 | 0.47 | 0.42 | 0.30 | 0.81 | 0.21 | 0.70 | 0.69 | 0.69 |
| Treatment Energy | 0.00 | 0.00 | 0.00 | 0.00 | 0.00 | 0.01 | 0.00 | 0.00 | 0.00 |
| Waste Sludge | 0.04 | 0.02 | 0.01 | 0.00 | 0.06 | 0.08 | 0.00 | 0.06 | 0.03 |
| Waste Concrete | 0.00 | 0.00 | 0.00 | 0.00 | 0.00 | 0.00 | 0.00 | 0.03 | 0.00 |
| Waste Plastics | 0.00 | 0.00 | 0.00 | 0.00 | 0.00 | 0.01 | 0.00 | 0.03 | 0.00 |

### 4.2 Economic Sensitivity

The sensitivity analysis for the present worth of cost (PWC) identified capital cost, analysis period, and design flow as the most critical factors influencing lifetime cost estimates. These three inputs consistently showed the highest sensitivity across all scenarios, confirming that long-term financial planning hinges on accurate infrastructure cost estimation and realistic flow projections (Table S6).

Capital costs were particularly sensitive in infrastructure-heavy scenarios such as AM (0.87), AMM (0.85), and AMB/AMC (0.81 each), emphasizing the need for detailed cost data during design and procurement. The analysis period, representing the project lifespan, showed the highest overall influence, up to 0.97 in AM, demonstrating that assumptions about how long a system would operate significantly shaped its cost-effectiveness. Closely tied to this was design flow, which ranged from 0.30 to 0.54 across scenarios. Since PWC was reported per cubic meter, flow assumptions directly affected cost normalization and comparative feasibility.

Table S6: Sensitivity factors for life cycle cost analysis. Dark red shades indicate highly sensitive inputs.

| **Inputs Varied (+/- 20%)** | **CF** | **LF** | **AM** | **AMB** | **AMI** | **AMP** | **AMM** | **AMC** | **AMA** |
| --- | --- | --- | --- | --- | --- | --- | --- | --- | --- |
| Capital Cost | 0.77 | 0.69 | 0.87 | 0.81 | 0.68 | 0.49 | 0.85 | 0.81 | 0.53 |
| Energy | 0.00 | 0.00 | 0.01 | 0.04 | 0.04 | 0.05 | 0.04 | 0.03 | 0.04 |
| Consumables | 0.00 | 0.09 | 0.02 | 0.02 | 0.16 | 0.30 | 0.03 | 0.03 | 0.33 |
| Labor | 0.14 | 0.09 | 0.05 | 0.03 | 0.03 | 0.02 | 0.03 | 0.04 | 0.03 |
| Other O&M | 0.09 | 0.13 | 0.05 | 0.10 | 0.09 | 0.13 | 0.05 | 0.09 | 0.12 |
| Benefits | 0.00 | 0.00 | 0.00 | 0.00 | 0.00 | 0.00 | 0.00 | 0.00 | 0.04 |
| Design Flow | 0.48 | 0.43 | 0.54 | 0.50 | 0.42 | 0.30 | 0.52 | 0.50 | 0.32 |
| Interest Rate | 0.11 | 0.15 | 0.06 | 0.09 | 0.15 | 0.25 | 0.08 | 0.09 | 0.23 |
| Analysis Period | 0.91 | 0.87 | 0.97 | 0.93 | 0.86 | 0.75 | 0.95 | 0.93 | 0.77 |

Operational cost components such as consumables were highly sensitive in scenarios with intensive chemical use, most notably AMA (0.33), AMP (0.30), and AMI (0.16). This suggested that variability in chemical dosing rates, prices, or supplier contracts could lead to large swings in long-term cost (Schrammel, 2015). Energy showed lower but non-negligible influence in energy-intensive scenarios like AMP and AMI (both 0.05), while labor and other O&M had moderate influence across most configurations. Interestingly, interest rate sensitivity was highest in AMP (0.25) and AMA (0.23), reflecting the compound impact of financing assumptions on capital-intensive systems (Ghangrekar, 2022). Although monetizable benefits (revenue from harvested algae) showed little sensitivity overall, AMA registered a minor effect (0.04), suggesting that even small offsets can become relevant in high-cost systems.

# Uncertainty

## 5.1 Environmental Uncertainty

Environmental impacts were evaluated using SimaPro 9.5 (PhD version) uncertainty feature, which applies a Monte Carlo simulation approach. For each treatment alternative, 1,000 iterations were conducted across three key impact categories: GWP; MEP; and FEP. All parameters were modelled using normal distributions, as the life cycle inventory data was provided in the form of minimum and maximum values (Chowdhury, 2012).

Simulation results showed that mean GWP values ranged from 0.942 to 2.98 kg CO₂ eq/m³, with coefficients of variation (CVs) between 7.02% and 14.1%, reflecting moderate relative uncertainty across alternatives. MEP values ranged from 0.00211 to 0.00903 kg N eq /m³, with CVs from 9.35% to 12.5%, while FEP values ranged from 0.00348 to 0.01381 kg P eq /m³, with CVs between 9.25% and 12.6%. The standard error of the mean (SEM) remained low across all categories, ranging from 6.2×10⁻⁶ to 1.3×10⁻², indicating high precision in the simulated outputs. Together, these results highlight both the magnitude of differences in environmental performance and the reliability of the estimates. In the graphical outputs, error bars representing standard deviations make these uncertainties explicit, allowing alternatives to be compared not only by central tendencies but also by the robustness of their ranges, an important consideration for informed and risk-aware decision-making (Salciccioli et al., 2016).

## 5.2 Economic Uncertainty

To account for variability and uncertainty in the economic analysis, a python-based Monte Carlo simulation was conducted using 100,000 iterations for each of the nine treatment alternatives. Cost-related parameters, including capital, operational, and maintenance costs, were modeled using lognormal distributions to reflect their positively skewed, non-negative nature (Bashar et al., 2018). The design flow and analysis period were assumed to follow normal distributions, representing symmetric uncertainty around expected values, while the interest rate was modeled using a uniform distribution to capture equal probability within a plausible range (Ghangrekar, Makarand M., 2022). For all distributions, bounds were set such that ±20% of the mean corresponded to three standard deviations, aligning with a 99.7% confidence interval and allowing for consistent modeling of variability (Chowdhury, 2012).

The results of the simulations yielded a range of present cost outcomes, from $0.206/m³ for CF to $1.193/m³ for AMP, with corresponding median, standard deviation, and 95% confidence intervals. Coefficients of variation (CVs) ranged from 6.86% to 9.39%, with AMP exhibiting the lowest relative variability and AM the highest. The 95% confidence intervals were narrowest for CF and LF and widest for AMP and AMA, suggesting higher cost predictability in simpler systems and greater uncertainty in more complex or capital-intensive options (Salciccioli et al., 2016). The standard error of the mean (SEM) was ≤ $0.0003 across all alternatives, indicating a high level of precision in the simulation outputs. This probabilistic framework provides a more nuanced understanding of cost performance under uncertainty and supports risk-informed decision-making for selecting appropriate treatment technologies. In the graphical outputs, error bars representing standard deviations make these uncertainties explicit, allowing alternatives to be compared not only by central tendencies but also by the robustness of their ranges. Detailed results tables are provided in the *Supplementary Tables 4 and 6.*

# References

Bashar, R., Gungor, K., Karthikeyan, K. G., & Barak, P. (2018). Cost effectiveness of phosphorus removal processes in municipal wastewater treatment. *Chemosphere*, *197*, 280–290. https://doi.org/10.1016/j.chemosphere.2017.12.169

Chowdhury, S. (2012). Decision making with uncertainty: An example of water treatment approach selection. *Water Quality Research Journal*, *47*(2), 153–165. https://doi.org/10.2166/wqrjc.2012.107

Cornejo, P. K., Zhang, Q., & Mihelcic, J. R. (2013). Quantifying benefits of resource recovery from sanitation provision in a developing world setting. *Journal of Environmental Management*, *131*, 7–15. https://doi.org/10.1016/j.jenvman.2013.09.043

Edelen, A., & Ingwersen, W. (2016). *Guidance on Data Quality Assessment for Life Cycle Inventory Data* (EPA/600/R-16/096; pp. 5–19). U.S. Environmental Protection Agency. https://nepis.epa.gov/Exe/ZyPURL.cgi?Dockey=P100R8JX.TXT

Ghangrekar, Makarand M.,. (2022). Life Cycle Costing of Wastewater Treatment. In *Wastewater to water: Principles, technologies and engineering design*. Springer Nature Singapore Pte Ltd. 2022. https://doi.org/10.1007/978-981-19-4048-4_21

Jaczynska, K., Ruto, D., Orner, K., & Mehta, S. (2025). A comparative life cycle assessment of textile fiber production processes: Hemp versus cotton. *Cleaner Waste Systems*, *11*, 100277. https://doi.org/10.1016/j.clwas.2025.100277

Salciccioli, J. D., Crutain, Y., Komorowski, M., & Marshall, D. C. (2016). Sensitivity Analysis and Model Validation. In Mit Critical Data, *Secondary Analysis of Electronic Health Records* (pp. 263–271). Springer International Publishing. https://doi.org/10.1007/978-3-319-43742-2_17

Schrammel, E. (2015). *A cost-benefit analysis of hydroponic wastewater treatment in Sweden*. https://api.semanticscholar.org/CorpusID:52841550

Weidema, B. P., & Wesnæs, M. S. (1996). Data quality management for life cycle inventories—An example of using data quality indicators. *Journal of Cleaner Production*, *4*(3–4), 167–174. https://doi.org/10.1016/S0959-6526(96)00043-1
